# Phytochemicals increase the antibacterial activity of antibiotics by acting on a drug efflux pump

**DOI:** 10.1002/mbo3.212

**Published:** 2014-09-16

**Authors:** Thelma Ohene-Agyei, Rumana Mowla, Taufiq Rahman, Henrietta Venter

**Affiliations:** 1 Department of Pharmacology, University of CambridgeTennis Court Road, Cambridge, CB2 1PD, United Kingdom; 2 School of Pharmacy & Medical Sciences, Sansom Institute for Health Research, University of South AustraliaGPO Box 2471, Adelaide, 5001, Australia

**Keywords:** Antibiotic, efflux pump inhibitors, multidrug resistance, natural products

## Abstract

Drug efflux pumps confer resistance upon bacteria to a wide range of antibiotics from various classes. The expression of efflux pumps are also implicated in virulence and biofilm formation. Moreover, organisms can only acquire resistance in the presence of active drug efflux pumps. Therefore, efflux pump inhibitors (EPIs) are attractive compounds to reverse multidrug resistance and to prevent the development of resistance in clinically relevant bacterial pathogens. We investigated the potential of pure compounds isolated from plants to act as EPIs. In silico screening was used to predict the bioactivity of plant compounds and to compare that with the known EPI, phe-arg-*β*-naphthylamide (PA*β*N). Subsequently, promising products have been tested for their ability to inhibit efflux. Plumbagin nordihydroguaretic acid (NDGA) and to a lesser degree shikonin, acted as sensitizers of drug-resistant bacteria to currently used antibiotics and were able to inhibit the efflux pump-mediated removal of substrate from cells. We demonstrated the feasibility of in silico screening to identify compounds that potentiate the action of antibiotics against drug-resistant strains and which might be potentially useful lead compounds for an EPI discovery program.

## Introduction

Antibiotic resistance is one of the world's most pressing health problems (Gootz 2010; Bush et al. 2011; Wise 2011; Piddock 2012). Paradoxically, the increase in antibiotic-resistant bacteria coincides with a dramatic reduction in the number of pharmaceutical companies developing new antimicrobial agents (Cooper and Shlaes 2011).

In Gram-negative pathogens, drug efflux protein complexes such as AcrAB-TolC confer resistance to antibiotics from many different classes and are the systems predominantly responsible for innate and acquired multidrug resistance (MDR) (Zhang et al. 2000; Poole 2001; Piddock 2006a, 2006b; Du et al. 2013). One way of prolonging antibiotic efficiency or of potentiating the activity of antibiotics against drug-resistant pathogens is by blocking drug efflux pumps with efflux pump inhibitors (EPIs) (Lomovskaya et al. 2006; Abreu et al. 2012; Kourtesi et al. 2013; Ruggerone et al. 2013). Inhibiting bacterial drug efflux machinery has additional benefits apart from the reversal of drug resistance because drug efflux pumps also have crucial roles in bacterial pathogenesis, virulence, and biofilm formation (Piddock 2006b; Martinez et al. 2009; Baugh et al. 2012, 2014). In addition, functional efflux pumps are necessary for the selection of drug-resistant bacteria (Lomovskaya and Bostian 2006; Ricci et al. 2006; Zhang et al. 2011).

A large proportion of the drugs used to treat infectious diseases have come from natural sources. Plants have evolved to effectively fight off infections by producing an array of special chemicals. We were specifically interested in identifying and developing EPIs from plant sources. So far, the plant compounds that have been shown to act synergistically with antibiotics were predominantly effective against Gram-positive bacteria (Lewis 2001; Stavri et al. 2007), while EPIs for Gram-negative pathogens are still evading us as phe-arg-*β*-naphthylamide (PA*β*N) and its derivatives are not clinically used due to problems with stability and toxicity (Marquez 2005). Gram-negative bacteria have evolved a significant permeability barrier to amphipathic compounds by the combination of an outer membrane and the expression of drug efflux pumps. Due to their intrinsic high drug resistance and the increased incidence of drug-resistant Gram-negative infections, it is important that new treatments against drug-resistant Gram-negative bacteria are developed.

In this respect, AcrB the inner membrane protein from the AcrAB-TolC drug transport complex is an ideal model for testing compounds with EPI properties. The structure of AcrB from *Escherichia coli* with and without substrates/inhibitors bound has been solved (Murakami et al. 2006; Seeger et al. 2006; Nakashima et al. 2011; Eicher et al. 2012; Hung et al. 2013). In addition, the first structure of a tripartite drug efflux complex has recently been published (Du et al. 2014).

Furthermore, AcrB homologues are present in most Gram-negative pathogens; it can even substitute for MexB from *Pseudomonas aeruginosa* and form an active drug efflux complex with MexA and OprM, the natural partners of MexB (Krishnamoorthy et al. 2008; Welch et al. 2010). Hence, an inhibitor of AcrB has great potential to be an EPI in other Gram-negative pathogenic organisms.

AcrB forms an asymmetric homotrimer where the monomers cycle through three conformational stages designated the loose, tight, and open or access, binding and extrusion stages according to Seeger et al. (Seeger et al. 2006) or Murakami et al. (Murakami et al. 2006), respectively. Structures with bound drugs revealed two discrete multisite binding pockets separated by a switch loop; the distal pocket in the binding/tight state and a proximal pocket in the access/loose state (Nakashima et al. 2011; Eicher et al. 2012).

A structure of AcrB with an inhibitor bound demonstrated that the inhibitor binding site partially overlaps with the minocycline-binding site in the distal binding pocket, while another part of the inhibitor binds to a narrow hydrophobic pit lined by several phenylalanine residues (F136, F178, F610, F615, F628). Tight binding of the inhibitor to residues in the pit prevents the functional rotation through the three stages; hence, prevents the efflux of antibiotics (Nakashima et al. 2013).

In this study, we used in silico screening to select five plant-derived compounds on their predicted ability to bind and inhibit AcrB. The selected compounds were screened for their antibacterial effect on wild-type *E. coli* cells and *E. coli* cells with a genomic deletion of the gene coding for AcrB. The ability of the compounds to improve antibiotic sensitivity to efflux-mediated-resistant *E. coli* cells was assessed. Direct inhibition of substrate transport was observed by measuring the reduction in Nile Red efflux in the presence of the natural products.

## Materials and Methods

### Bacterial strains, media, and chemicals

Bacteria used in this study were *E. coli* strain BW25113 wild type and strain BW25113 with a deletion of *acrB* (a gift from Professor Martin Pos, Institute of Biochemistry, Goethe-University, Frankfurt am Main, Germany). All strains were maintained in 25% (v/v) glycerol at −80°C until required. LB media were obtained from Acumedia and Agar from Oxoid. All antibiotics, chemicals, and natural products were obtained from Sigma.

### Accumulation of fluorescent compounds

LB-Broth was inoculated with an overnight culture of wild-type-resistant *E. coli* cells or cells with a genomic deletion of *acrB* (1:500 dilution) and incubated with shaking at 37°C until an OD_660_ of 0.5 was reached. The cells were harvested by centrifugation at 3000*g* for 10 min at 4°C, then washed three times by resuspension in 50 mmol/L potassium phosphate buffer (pH 7.0) containing 5 mmol/L MgSO_4_, and sedimented by centrifugation at 3000 *g* for 10 min at 4°C. The cells were then resuspended in the same buffer to an OD_660_ of 0.5 and incubated for 3 min at room temperature (25°C) in the presence of 25 mmol/L glucose to energize or 10* μ*mol/L carbonyl cyanide 3-chlorophenylhydrazone (CCCP) to deenergize the cells. The fluorescence measurement was started, and 60 sec later 2 *μ*mol/L ethidium bromide, 0.125 *μ*mol/L Hoechst 33342, or 0.25 *μ*mol/L TMA-DPH [1-(4-trimethylammoniumphenyl)-6-phenyl-1,3,5-hexatriene *p-*toluenesulfonate] was added. The fluorescence was followed as a function of time in a PerkinElmer, Beaconsfield UK, LS 55B fluorimeter. Excitation and emission wavelengths and excitation and emission slit widths were 500, 580, 5, and 10 nm, respectively, for ethidium bromide; 355, 457, 10, and 4 nm, respectively, for Hoechst 33342; and 350, 425, 5, and 5 nm, respectively, for TMA-DPH.

### Docking of natural products to AcrB

Docking of natural products was performed using the *E. coli* AcrB protein (Protein Data Bank: 4DX5, tight monomer) and AutoDock 4.2 (http://autodock.scripps.edu/) (Morris et al. 2009). Prior to docking, bound ligand (minocycline) was removed and the suitability of AutoDock was validated by blind cognate docking (Hetenyi and van der Spoel 2011). For this, the bound minocycline we first removed from the reference structure and minocycline (the structure of which was obtained from a separate source – the Zinc database, https://docking.org/) was redocked against the entire protein structure. The top-ranked pose of redocked minocycline largely superimposed (within ≤2Å) over the original pose of the drug present in the crystal structure (not shown). Since the putative interaction site(s) for the screened natural products were unknown, we used blind docking approach where the search space for the ligands comprised a grid that contained the entire protein structure. Ligands were downloaded from PubChem database (http://pubchem.ncbi.nlm.nih.gov/) and were energy minimized with MM2 force field using ChemBioOffice 2008 (http://www.cambridgesoft.com). Polar hydrogens and the Gasteiger partial atomic charges were then added to the protein and ligands using AutoDockTools (http://autodock.scripps.edu/resources/adt), and the prepared structures were used as input files for docking. From the estimated free energy of ligand interaction (ΔG_binding_, kcal/mol), the inhibition constants (Ki) were calculated (Musa et al. 2011). Only the best pose (the one with the lowest ΔG_binding_) is considered. Finally, the best ligand poses were analyzed using PoseViewWeb (http://poseview.zbh.uni-hamburg.de/). PyMol was used for figures.

### Antimicrobial assays

Antimicrobial assays were carried out according to the 96-well microtiter broth dilution method (Ohene-Agyei et al. 2012). Briefly, the test compounds were added to the cell suspensions (10^5^ cells/well in LB-broth) at increasing concentrations, and the cultures were incubated at 37°C with shaking. The A_630_ of the cultures were measured in a plate reader (BioTek, Potton, Bedfordshire, UK) after 18 h, and the lowest concentration of drug needed to prevent growth (no increase in A_630_ compared to the A_630_ at time zero) was determined by minimum inhibitory concentrations (MIC). Additionally, for some compounds where MIC determination with the plate reader was impaired due to their high optical densities bacterial growth was also determined using resazurin where changes of the color from blue to pink indicated a reduction of the compound caused by bacterial growth (Ndi et al. 2007). From this the MIC were determined as the lowest concentration of plant extract where no growth was observed (color remained blue).

All experiments employed basal levels of expression without induction. All MIC determinations were repeated at least three times in independent experiments. The antibiotics, compounds, and EPI were made up and used according to the manufacturer's instructions.

### Checkerboard titration assay method

Interactions between antibiotics and natural products were assessed by a checkerboard titration assay (Lomovskaya et al. 2001; Orhan et al. 2005). The antibiotic was tested at seven concentrations, whereas the natural product was tested at five concentrations. A quantity of 100 *μ*L of LB-broth was distributed into each well of the microdilution plates. The antibiotic was serially diluted along the ordinate, while the natural product was diluted along the abscissa. Each microtiter well was then inoculated with 100 *μ*L of a bacterial inoculum to give 10^5^cells/well. The plates were incubated at 37°C for 7 h under aerobic conditions.

The MIC were defined as the lowest concentration of antibiotic that completely inhibited the growth of the organism as detected by no increase in A_630_ over the 7 h. This was also confirmed with the use of resazurin to test cell viability (Ndi et al. 2007).

### Nile red efflux assay

The ability of the natural products to reduce the efflux of efflux pump substrates was assessed by measuring the reduction in Nile Red efflux (Bohnert et al. 2010). LB-Broth (Acumedia from Cell Biosciences, Heidelberg, Victoria, Australia) was inoculated with an overnight culture of the required *E. coli* strain and incubated with shaking at 37°C until the OD_660_ did not increase any more. The cells were harvested by centrifugation at 3000*g* for 10 min at room temperature, then washed three times by resuspension in 50 mmol/L potassium phosphate buffer (pH 7.0) containing 1 mmol/L MgCl_2_ and sedimented by centrifugation at 3000*g* for 10 min at 4°C. The cells were then resuspended in the same buffer to an OD_660_ of 1 after which cells were allowed to rest for 15 min at room temperature. Aliquots (2 mL) were then transferred to Pyrex 15-mL conical centrifugation tubes and CCCP was added to a final concentration of 10 *μ*mol/L for the AcrAB-overexpressing strain. After 15 min, the natural product was added to the desired final concentration and Nile Red added to a final concentration of 5 *μ*mol/L after another 15 min. The cell suspension was incubated on a shaker (140 rpm; 37°C) for 3 h. The cells were left at room temperature for 60 min and then centrifuged for 5 min at 3000*g*. After the bulk of the supernatant was discarded, droplets of it clinging to the tube wall were thoroughly removed with absorbent tissues, and the cells were resuspended in 2-mL assay buffer. Immediately thereafter, 0.2 mL of this cell suspension was transferred to a quartz cuvette (PerkinElmer, UK) containing 1.8 mL of assay buffer, and the cuvette was placed in a PerkinElmer LS 55B fluorimeter. The excitation was at 552 nm, emission was at 636 nm, and excitation and emission slit widths were 10 and 5 nm, respectively. The fluorescence of the cell suspension was followed for 100 sec, after which Nile Red efflux was triggered by rapid energization with 50 mmol/L glucose. Fluorescence was monitored for another 200 sec.

### Nitrocefin uptake assay

Nitrocefin uptake assay was carried out as described previously (Lomovskaya et al. 2001). *E. coli* cells with constitutive expression of chromosomal *β*-lactamase were grown in LB-broth, harvested, and washed in 50 mmol/L Potassium phosphate buffer. The cells were subsequently resuspended in the same buffer to OD_660_ of 0.5. The cell suspension was treated with 10 *μ*mol/L CCCP and the plant compounds added to give the required final concentrations. Nitrocefin was then added to give a final concentration of 32 *μ*g/mL. Hydrolysis of nitrocefin was monitored by measuring the increase in absorbance at 490 nm with a plate reader (BioTek).

## Results and Discussion

### Strains used in this study

As we wanted to identify plant products that could act as EPIs, it was important to distinguish between inhibition of AcrB-mediated antibiotic efflux and altered membrane permeability. In order to do this, we have compared the resistance profiles of wild type, drug-resistant *E. coli* cells with cells where the gene coding for the drug efflux protein AcrB has been deleted. We have used the uptake of fluorescent substrates to show that there is no intrinsic difference between the *E. coli* strains used in this study and that any effect observed would not be due to intrinsic differences in cell permeability.

**Figure 1:**
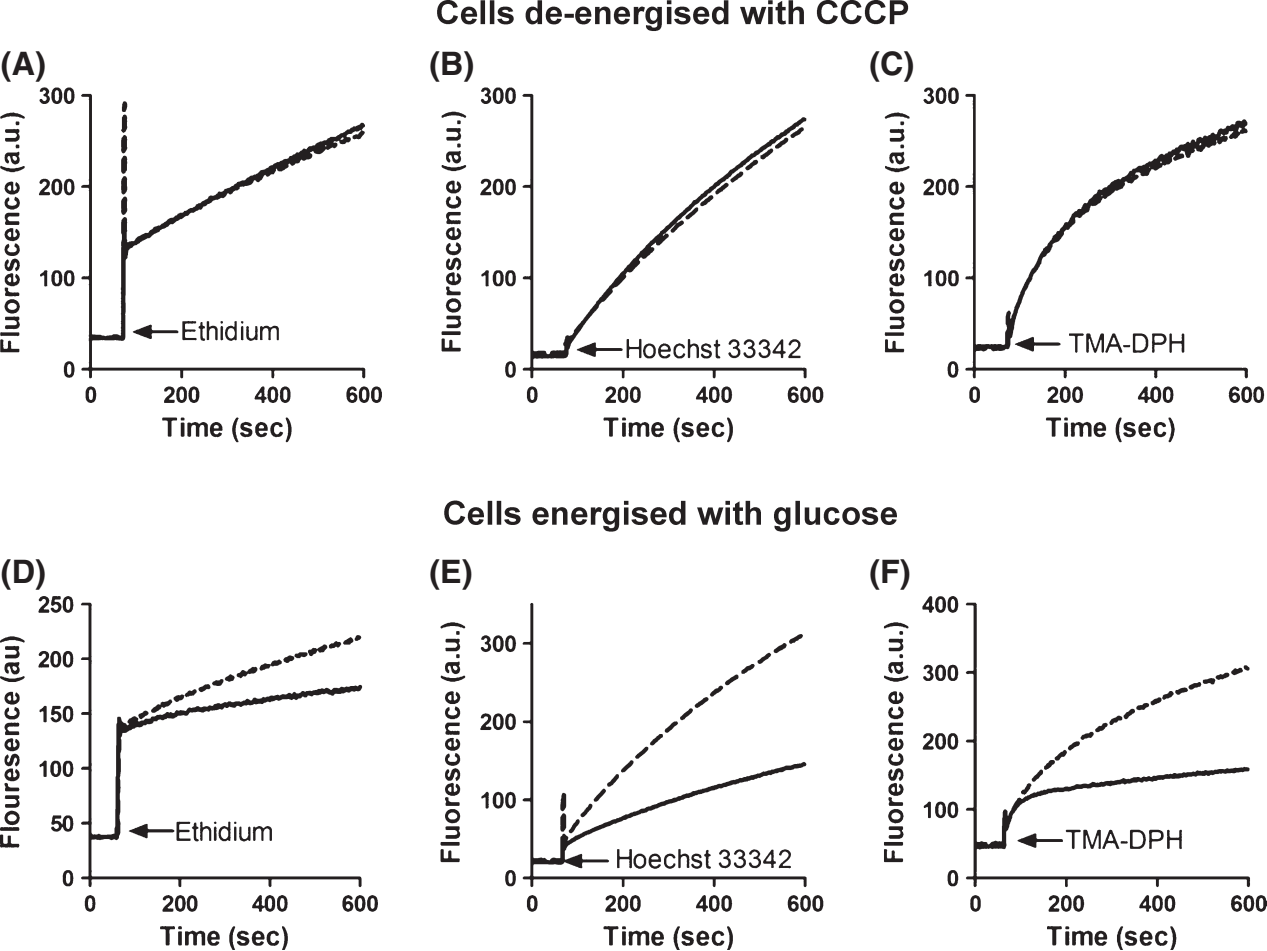
Accumulation of fluorescent compounds in the *Escherichia coli* strains used in this study. *E. coli* BW25113 (black line) and BW25113 ΔAcrB (broken line) cells were grown to an OD_660_ of 0.5. Cells were harvested, washed three times in 50 mmol/L potassium phosphate buffer (pH 7.0) containing 5 mmol/L MgSO_4_ and resuspended to a final OD_660_ of 0.5. (A–C). Cells were treated with carbonyl cyanide 3-chlorophenylhydrazone (CCCP) (10 *μ*mol/L) for complete deenergization. (D–F). Cells were incubated in the presence of glucose (20 mmol/L) to generate metabolic energy before the fluorescence trace was started. Ethidium (2 *μ*mol/L), Hoechst 33342 (0.125 *μ*mol/L), or TMA-DPH (0.25 *μ*mol/L) were added at the points indicated by the arrows and the increase in fluorescence was followed over time.

Ethidium, Hoechst 33342 and TMA-DPH all share the property that they are virtually nonfluorescent in aqueous solution while undergoing a large increase in fluorescent quantum yield when in a hydrophobic environment such as when in a lipid bilayer or intercalated into DNA. Hence, the passive permeation of these compounds into bacterial cells can be followed by the increase in their fluorescence. It is clear from Figure 1A–C that the fluorescence (and hence passive permeation) of all three fluorescent compounds are identical in *E. coli* cells which have been deenergized by the addition of the protonophore CCCP. Therefore, there is no intrinsic difference in permeability between the cells used in this study. However, when the cells are energized by the addition of glucose, there is a marked decrease in fluorescence between the wild type (resistant) and ΔAcrB cells (Fig. 1D–F). This decrease in fluorescence is representative of AcrB-mediated efflux of the fluorescent compounds. Combined, the results mean that any effects on the MICs for the test compounds observed would be due to the active AcrB-mediated efflux of the compounds and not due to intrinsic differences in the membrane permeability between the strains used in the study.

### Plant compounds studied

**Figure 2:**
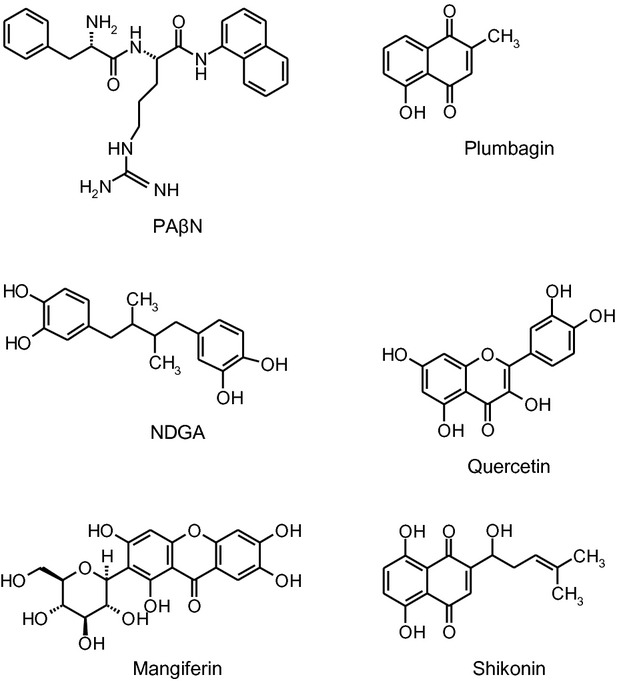
Structures of PA*β*N and the phytochemicals.

In silico screening of an in-house database of 50 phytochemicals identified five different compounds that could be docked in the binding pocket of AcrB similar to the known EPI PA*β*N. These were the naphthoquinones plumbagin from *Plumbago indica* and shikonin from *Lithospermum erythrorhizon*; the flavonoid quercetin which is found in many fruits and vegetables; the xanthonoid mangiferin from *Mangifera indica* (mango), and the antioxidant nordihydroguaretic acid (NDGA) from creosote bush (Fig. 2).

Plumbagin has previously been reported as an antibacterial compound which became more potent in the presence of the EPI PA*β*N (Tegos et al. 2002; Kuete et al. 2011). In addition, plumbagin is also an inhibitor of ABCG2, the ATP-binding cassette transporter involved in the multidrug-resistant phenotype of cancer cells (Shukla et al. 2007). Many compounds inhibit drug transporters from both cancer cells and bacteria (Amaral et al. 2012) as the drug efflux proteins involved display similarities in their mechanism of action (Venter et al. 2003, 2005). Quercetin has previously been reported as a substrate for AcrB as deletion of AcrB from *E. coli* resulted in a more than 8-fold reduction in the MIC of quercetin (Al-Karablieh et al. 2009). Nordihydroguaretic acid is bioactive compound from creosote bush, a plant which has long been used for its medicinal properties by North American Indians (Ross 2005) while antimicrobial effects of NDGA have recently been reported (Martins et al. 2013). Mangiferin has many medicinal qualities (Matkowski et al. 2013) and limited antibacterial (Savikin et al. 2009) and biofilm inhibitory (Annapoorani et al. 2012) effects have also been reported. Shikonin is a compound isolated from herbs used in traditional Chinese medicine (Tse 2003; Andújar et al. 2013). It has antibacterial activity against Gram-positive organisms (Shen et al. 2002; Haghbeen et al. 2011) but not against Gram-negative bacteria such as *P. aeruginosa* and *E. coli* (Shen et al. 2002; Haghbeen et al. 2011).

### In silico prediction of the binding of PA*β*N and phytochemicals to AcrB

**Figure 3:**
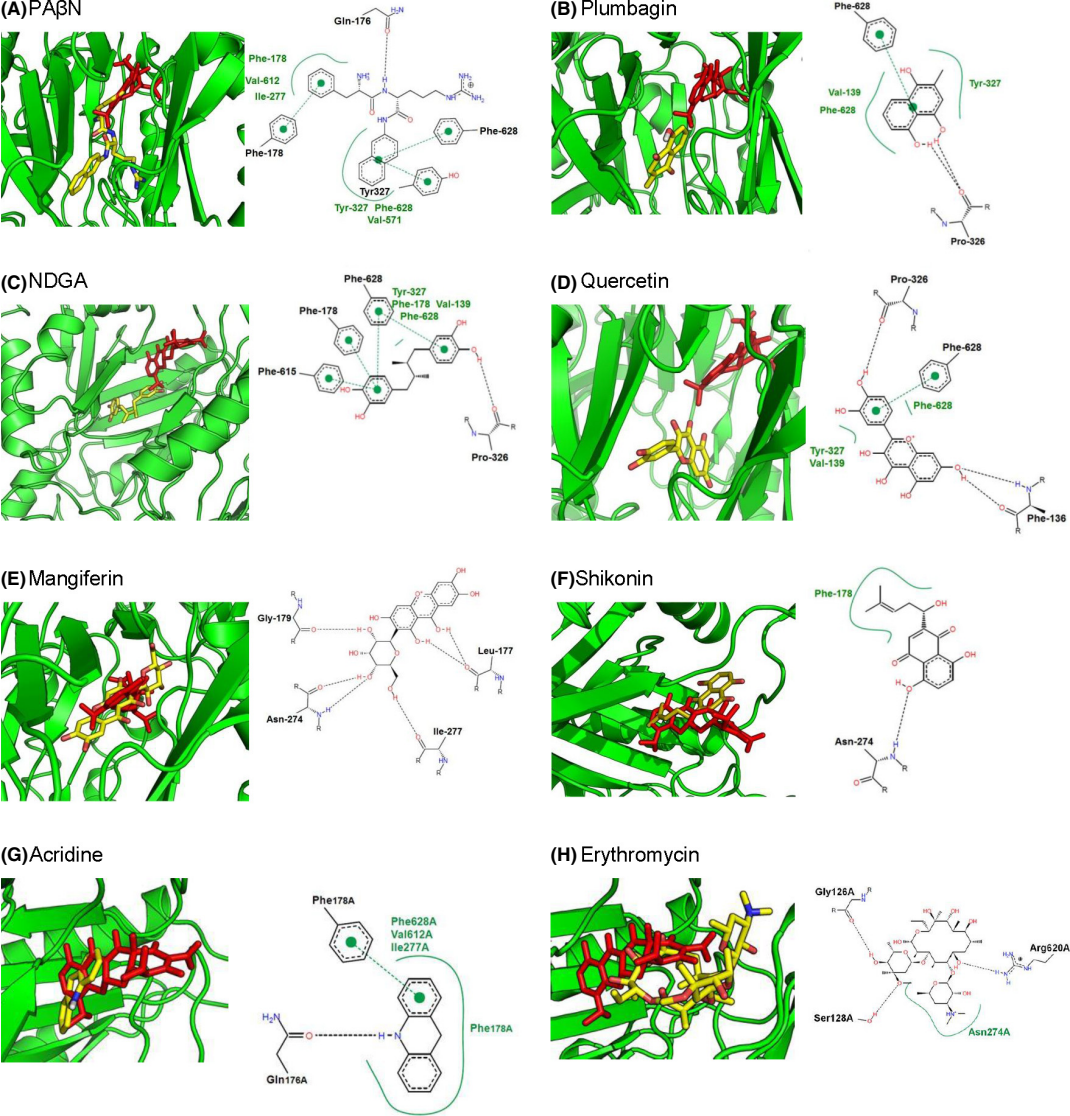
Docking of phytochemicals and substrates onto AcrB (PDB: 4DX5, tight monomer). Positions of the examined ligands (yellow) compared to minocycline (red) are given in the left-hand pictures, while the interaction of the amino acids with the ligands is given on the right-hand side in each picture.

The binding of natural products to AcrB was predicted through docking experiments and compared with the docking of the known inhibitor PA*β*N and substrate minocycline. The T (tight or binding) monomer of AcrB (PDB code 4DX5) (Eicher et al. 2012) with minocycline bound to the deep binding pocket was used in the docking experiments. PA*β*N docked in a position closer to the lateral binding site of the T monomer compared to minocycline (Fig. 3A) and is stabilized by interactions with amino acids Phe-628, Phe-178, Tyr-287, and a hydrogen bond to the oxygen of Gln176 (Fig. 3A). The predicted docking site of PA*β*N correlates well with the binding of PA*β*N to AcrB as predicted by dynamic simulations where Phe-628, Phe-178, and Gln-176 were also some key residues for interaction with PA*β*N (Vargiu and Nikaido 2012). In addition, a recent crystal structure of AcrB and MexB revealed that Phe 178 contributes to the tight binding of an inhibitor molecule through *π*–*π* interactions (Nakashima et al. 2013). Incidentally, all the natural products which were predicted to be potential EPIs docked in a similar position as PA*β*N, while substrates such as minocycline, acridine, and erythromycin docked in the deep binding pocket (Fig. 3). Vargiu and Nikaido (Vargiu and Nikaido 2012) utilized molecular dynamics simulations which also predicted that residues from the deep binding pocket were much more involved in interaction with a range of substrates, while less involved in stabilizing the inhibitor PA*β*N. In our in silico binding predictions, all natural products were stabilized by hydrogen bonding and extensive hydrophobic interactions between phenylalanine side chains and aromatic groups within the compounds. This is not surprising as phenylalanine residues play an important role in substrate recognition and stabilization in RND-type drug transporters (Ohene-Agyei et al. 2012). According to the docking, mangiferin would act more like a substrate than an inhibitor and the predicted binding affinity was also the lowest of the natural products included in the assays. The free energy of interaction (ΔG_binding_) determined from the docking of each compound was used to predict the K_i_ for the interactions (Table 1). Accordingly, the compounds could be arranged into an order of putative inhibition efficiency given as:

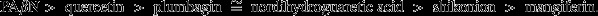


**Table 1:** Free energy of binding and affinity constants calculated from the docking experiments

Ligand	ΔG_binding_ (kcal/mol)	Predicted K_i_ (*μ*mol/L)
PA*β*N	−7.95	1.5
Plumbagin	−6.7	12.3
NDGA	−6.66	13.1
Quercetin	−7.15	5.7
Mangiferin	−5.56	84
Shikonin	−6.02	38.6
Acridine	−7.8	1.9
Erythromycin	−8.6	0.5

Subsequently, bioassays were performed to determine the validity of the in silico screening/modeling data.

### Antibacterial activity of the plant compounds

The MIC of the natural products were determined so that subinhibitory concentrations could be used to check for efflux pump inhibition. For this purpose, wild-type-resistant *E. coli* BW25113 cells or cells with a genomic deletion of *acrB* were used to test the antibacterial activity of the natural products. Cells were grown in the presence of increasing concentrations of PA*β*N or in the presence of increasing concentrations of natural products. PA*β*N is toxic to cells at high concentrations and the presence of AcrB confers resistance to the cells against PA*β*N in accordance with the well-established role of PA*β*N as an EPI that is also an efflux pump substrate (Table 2).

**Table 2:** Minimum inhibitory concentrations (MICs) of natural products and PA*β*N against *Escherichia coli* strains

*E. coli* strain	MIC (mg/L)					
	Plumbagin	NDGA	Quercetin	Mangiferin	Shikonin	PA*β*N
ΔAcrB drug-sensitive strain	16	64	>1024	>512	>256	128
Wild-type drug-resistant strain	128	512	>1024	>512	>256	512

Of the compounds tested, only plumbagin and NDGA were antibacterial with a significant increase in the levels of susceptibility for the strains with the *acrB* deletion. This indicates the role of the AcrAB-TolC efflux pump in recognizing and transporting plumbagin and NDGA (Table 2). Quercetin, shikonin, and mangiferin were not toxic to bacterial cells at the maximum concentrations tested (Table 2).

According to our docking data, quercetin should be the best inhibitor of AcrB after PA*β*N. Cells lacking AcrB have also been shown to be hypersensitive to quercetin (Al-Karablieh et al. 2009). However, the *E. coli* strain we used was not susceptible to quercetin.

In order for an compound to qualify as an EPI, it must be able to potentiate the effect of antibiotics which are effluxed in strains containing an efflux pump and must not potentiate the activities of antibiotics that are not effluxed (Lomovskaya et al. 2001). We therefore tested the ability of the natural products to reverse drug resistance in AcrB expressing cells.

### Some natural products are able to increase drug sensitivity in drug-resistant cells

The ability of the compounds to reverse antibiotic resistance conferred by the expression of the AcrAB-TolC efflux pump was determined. Standard checkerboard assays in which the MIC of the antibiotics were determined in the presence of different concentrations of the known EPI PA*β*N and the natural products were performed (Table 3). PA*β*N was synergistic with erythromycin, chloramphenicol, novobiocin, and tetracycline. NDGA was the most efficient potential EPI as it synergized with erythromycin, chloramphenicol, novobiocin, tetracycline, and Tetraphenylphosphonium (TPP) (Table 3). Plumbagin increased sensitivity to erythromycin, chloramphenicol, and TPP, while shikonin potentiated the antimicrobial activity of TPP (Table 3). Plumbagin, NDGA, and shikonin had no effect of the MIC on the tested antibiotics against the AcrB null strain, which confirmed that the observed synergism for the wild-type cells is specific to AcrB inhibition (Table 4). Quercetin and mangiferin failed to synergize with any of the antibiotics tested (results not shown). We also tested the ability of the natural products to synergize with fluoroquinolones such as levofloxacin and ciprofloxacin, but failed to observe any synergism with these antibiotics.

**Table 3:** Minimum inhibitory concentration in the absence or presence of PA*β*N and natural products showing activity of natural products with different antibiotics and TPP against the drug-resistant (wild-type) strain of *Escherichia coli*

	MIC (mg/L)															
	PA*β*N (mg/L)				Plumbagin (mg/L)				NDGA (mg/L)				Shikonin (mg/L)			
	0	50	100	200	0	16	32	64	0	64	128	256	0	64	128	256
Erythromycin	256	128	64	32	256	256	128	64	256	256	256	64	256	256	256	256
Chloramphenicol	4	2	2	2	4	4	2	2	4	4	2	1	4	4	4	4
Novobiocin	512	256	128	128	512	512	512	512	512	512	512	64	512	512	512	512
TPP	1024	512	512	512	1024	512	512	256	1024	1024	128	64	1024	512	512	64
Tetracycline	1	1	1	0.5	1	1	1	2	1	1	1	0.25	1	1	1	1
Nalidixic acid	2	0.5	0.5	0.5	2	2	2	2	2	2	2	2	2	2	2	2

**Table 4:** Minimum inhibitory concentration in the absence or presence of PA*β*N and natural products showing activity of natural products with different antibiotics and TPP against the drug-sensitive (ΔAcrB) strain of *Escherichia coli*

	MIC (mg/L)				
	No drug	PA*β*N (50 mg/L)	Plumbagin (10 mg/L)	NDGA (25 mg/L)	Shikonin (256 mg/L)
Erythromycin	16	16	16	16	16
Chloramphenicol	1	1	1	1	1
Novobiocin	16	16	16	16	16
TPP	16	16	16	16	16
Tetracycline	0.5	0.5	0.5	0.5	0.5
Nalidixic acid	1	1	1	1	1

The fact that quercetin was unable to reverse the resistance of AcrAB-TolC-expressing cells against the antibiotics tested was unexpected as our docking data indicated that quercetin should be the best inhibitor of AcrB after PA*β*N. Quercetin has also been identified as a high-affinity substrate for TtgR the transcriptional repressor of TtgABC a major efflux pump of *Pseudomonas putida* (Alguel et al. 2007). The result obtained for the checkerboard assays can be interpreted in two ways, (1) quercetin could be a substrate, with no EPI activity or, (2) quercetin is unable to overcome the membrane permeability barrier of Gram-negative organisms and hence cannot access AcrB.

Further investigation would be needed to confirm which of the explanations apply. It is also worth to note that PA*β*N cannot reverse drug resistance for all drugs and has, for instance, no effect on efflux pump-mediated resistance to the dye ethidium (Lomovskaya et al. 2001). It also seems that similar to PA*β*N (Lomovskaya and Bostian 2006) the natural products act as substrates that compete with antibiotics and in this way reverse drug resistance. They are thus not trapping the efflux pump in an inactive conformation, but rather acting as high-affinity substrate inhibitors and would also explain why many inhibitors only synergize with some antibiotics.

### The natural products do not permeabilize the outer membrane

In order to rule out false positives, we subsequently verified that the observed reduction in MIC in the presence of the natural products was not a result of nonspecific mechanisms such as increased membrane permeability. This was done in two ways: firstly by ascertaining that the natural products did not potentiate the activities of antibiotics that are not effluxed and secondly by determining the outer membrane permeability in the presence of the natural products using nicrocefin (Lomovskaya et al. 2001). Rifampicin is not effluxed by AcrB (MIC for wild type and ΔAcrB strains of 16 mg/L). We therefore determined the effect of the natural products at the maximum concentrations used in Table 1 on the MIC of rifampicin. With the exception of PA*β*N, known for its outer membrane permeability properties where the MIC was reduced from 16 mg/L to 0.5 mg/L, none of the natural products affected the MIC of rifampicin (results not shown).

**Figure 4:**
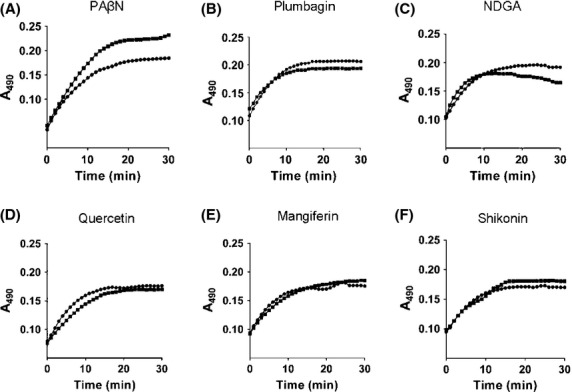
The phytochemicals do not permeabilize the outer membrane. The permeabilizing activity of compounds was measured as an increase in initial rates of nitrocefin hydrolysis by *Escherichia coli* wild-type cells treated with 10 *μ*mol/L CCCP. Dots represent no treatment and squares represent treatment with compounds at (A) PA*β*N at 128 *μ*g/mL, (B) plumbagin at 100 *μ*g/mL, (C) NDGA at 100 *μ*g/mL, (D) quercetin at 100 *μ*g/mL, (E) mangiferin at 500 *μ*g/mL, and (F) shikonin at 250 *μ*g/mL. Nitrocefin hydrolysis was monitored over time by monitoring the increase in absorbance at 490 nm. Representative fluorescent traces are shown for experiments with different cells batches done on three different days.

The outer membrane permeabilizing effect of the natural products was assessed by examining the rate of hydrolysis of a chromogenic *β*-lactam, nitrocefin by intact cells of *E. coli*. Hydrolysis of nitrocefin by the *β*-lactamase releases a colored compound that can be measured at 490 nm. The rate of nitrocefin hydrolysis is limited by the rate of diffusion across the outer membrane, hence an increased rate of hydrolysis of nitrocefin would be indicative of outer membrane permeabilization (Lomovskaya et al. 2001). The effect of the natural products on the rate of nitrocefin hydrolysis was determined at concentrations, where they did not interfere with the A_490_ measurements. We observed no increase in the rate of nitrocefin hydrolysis in the presence of the natural products. We could therefore conclude that the synergism with known antibiotics observed was not due to secondary mechanisms such as membrane permeabilization (Fig. 4).

### The natural products inhibit drug transport

**Figure 5:**
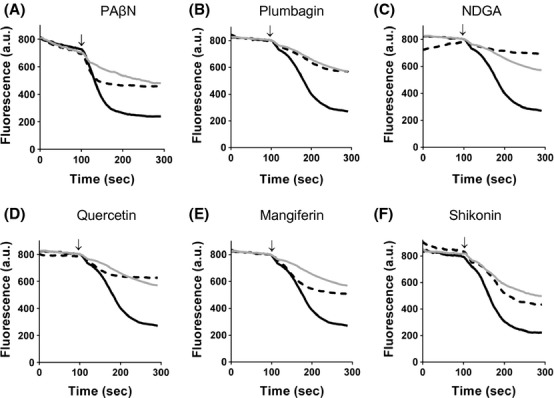
Inhibition of Nile Red efflux by the natural products. Wild-type-resistant cells (black line), ΔAcrB drug-sensitive cells (gray line) or wild-type cells in the presence of the natural products (dashed line) with (A) PA*β*N at 200 *μ*mol/L, (B) plumbagin at 50 *μ*mol/L, (C) NDGA at 100 *μ*mol/L, (D) quercetin at 200 *μ*mol/L, (E) mangiferin at 500 *μ*mol/L, and (f) shikonin at 25 *μ*mol/L were preloaded with Nile Red before the start of fluorescence measurements. Efflux was triggered at 100 sec by the addition of 50 mmol/L glucose (indicated by arrow). Representative fluorescent traces are shown for experiments with different cells batches done on three different days.

An EPI must also be able to reduce the level of extrusion of efflux pump substrates (Lomovskaya et al. 2001). We used the inhibition of Nile Red efflux to determine the ability of the natural products to reduce efflux pump activity (Bohnert et al. 2010). Nile Red is only weakly fluorescent in aqueous solutions, but undergoes a significant increase in fluorescent quantum yield when in nonpolar environments such as the cell membrane. Cells were preloaded with Nile Red in the absence or presence of the natural products. Once the cells were energized by the addition of glucose, the efflux of Nile Red could be observed as a drop in fluorescence. All the compounds tested were able to inhibit Nile Red efflux to an extent (Fig. 5). Plumbagin was the most efficient and complete inhibition of Nile Red efflux was observed using 50 *μ*mol/L of plumbagin. At much higher concentrations (500 *μ*mol/L), mangiferin was also able to inhibit the efflux, presumably through competition for the binding site, which would again be supportive of mangiferin being a low-affinity substrate rather than an inhibitor (Fig. 5).

## Conclusion

The obtained results of bioassays showed a good correlation with the molecular docking analysis, indicating that in silico screening could be a powerful tool to preselect compounds with potential EPI activity. To our knowledge, this is the first report of potential EPIs being identified with in silico screening and verified by bioassays.

Plumbagin, NDGA, and shikonin were able to increase susceptibility to antibiotics and toxic compounds and were also the most efficient in inhibiting AcrB-mediated substrate efflux; hence, they may be good lead compounds for a future drug discovery program. Given the similarity between AcrAB-TolC and the MexAB-OprM drug efflux system from pathogenic *P. aeruginosa*, future studies on MexAB-OprM using in silico screening and the methods described here have a high potential to deliver tangible outcomes in stemming the tide of drug-resistant infections.
